# Ekbom Syndrome Management in Elderly Patients: Challenges in Risperidone Titration and Treatment Adherence

**DOI:** 10.3390/pharmacy13020043

**Published:** 2025-03-16

**Authors:** Florina Madalina Mindru, Adrian Gheorghe Bumbu, Darian Faur

**Affiliations:** 1 Doctoral School of Biomedical Sciences, University of Oradea, 410087 Oradea, Romania; 2 Department of Psycho-Neuroscience and Recovery, Faculty of Medicine and Pharmacy, University of Oradea, 410073 Oradea, Romania; 3 Department of Psychology, West University of Timisoara, 300223 Timisoara, Romania; darian.faur99@e-uvt.ro

**Keywords:** Ekbom Syndrome, Delusional Parasitosis, Risperidone, antipsychotic therapy, treatment adherence, elderly patients

## Abstract

Ekbom Syndrome, also known as Delusional Parasitosis (DP), is considered a rare psychiatric condition. Based on diagnostic criteria, it is characterized by the strong belief of being infested with various parasites, as well as the presence of perceptual disturbances, usually tactile and/or visual hallucinations. The syndrome can manifest idiopathically or in connection with other medical conditions and substance use. Diagnosis is challenging, as patients tend to pursue dermatological care initially. This case report describes an 81-year-old female diagnosed with Ekbom Syndrome, presenting with severe anxiety, insomnia, and persistent delusions of infestation. Initial treatment with low-dose Risperidone (2 mg/day) was ineffective, requiring a dose escalation to 4 mg/day. However, the patient’s nonadherence to follow-up limited the assessment of long-term outcomes. This case highlights key clinical challenges in elderly patients, particularly dose titration, treatment response, and adherence issues. Comparative analysis with prior case reports suggests that higher doses of Risperidone (3–6 mg/day) may be required for symptom remission, but long-term outcomes remain uncertain. Additionally, nonadherence remains a major barrier, underscoring the need for structured monitoring and caregiver involvement. These findings offer insights into antipsychotic strategies for Ekbom Syndrome, highlighting individualized pharmacotherapy, long-term follow-up, and adherence support in elderly patients.

## 1. Introduction

Ekbom Syndrome can be classified as either primary or secondary. For the primary form, the delusion emerges spontaneously as a mono-delusional disorder, meeting the criteria outlined in the 10th revision of the International Classification of Diseases (ICD-10) for persistent delusional disorder, as well as the criteria in the 5th edition of the Diagnostic and Statistical Manual of Mental Disorders (DSM-5) for delusional disorder somatic type. The secondary form occurs as a result of another underlying medical condition. Commonly associated conditions include schizophrenia, dementia, depression, diabetes, neuropathies, and cardiovascular disorders. Additionally, DP can occur temporarily due to intoxication with substances such as amphetamines or cocaine or as an adverse effect of certain medications [1].

In clinical practice, second-generation antipsychotics such as Risperidone or Olanzapine are generally considered first-line treatments for Delusional Parasitosis, largely due to their safer profiles and better tolerability [2]. However, there are still insufficient data to establish a definitive gold standard for treatment. Over time, Pimozide, previously used as a first-line option, has fallen out of favor due to concerns regarding its safety profile, including QTc interval prolongation and a high risk of extrapyramidal symptoms [3]. When selecting an antipsychotic, clinicians must carefully evaluate both efficacy and potential side effects. It is important to note that all atypical antipsychotics carry the risk of metabolic dysfunction, necessitating regular monitoring of laboratory parameters during treatment. While recommended dosages range from 1 to 8 mg/day for Risperidone and 5 to 10 mg/day for Olanzapine, there is no established consensus on optimal dosing for Delusional Parasitosis. In elderly patients, dosing must be carefully adjusted based on age, kidney, and liver function and the presence of comorbidities [2]. Among other antipsychotic options, Haloperidol and Sulpiride are regarded as safer options. In cases where adherence to oral medication is a concern, depot antipsychotics may be considered as an alternative [4,5].

An increasing number of studies in the medical literature indicate that patients with Ekbom Syndrome are being treated more frequently with antidepressants, either as part of a combined therapy approach or as a standalone treatment. Selective serotonin reuptake inhibitors (SSRIs), in particular, have shown promise. In some instances, they are used to manage coexisting depressive symptoms, while in others, they serve as a monotherapy, resulting in a notable reduction in the symptoms of Ekbom Syndrome [6,7].

In this case report, we present the case of a patient and the challenges encountered in managing her treatment and achieving symptom remission. Particular emphasis was placed on treatment with Risperidone, highlighting both its role as a primary therapeutic option and the limitations observed in its efficacy.

## 2. Case Presentation

An 81-year-old woman was admitted to the psychiatric service and brought to the hospital by ambulance from her home, following a request from her family, due to severe anxiety, insomnia, and complaints of a severe infestation of her body, house, and belongings with worms. She reported experiencing tactile and proprioceptive hallucinations, describing seeing worms and worm eggs on her body and surroundings and feeling the worms moving beneath her skin.

The symptoms of worm infestation had been ongoing for approximately a year and a half. The issue began when the patient believed she was suffering from a parasitic infestation, prompting her to undergo numerous stool sample tests, all of which returned negative results. Despite this, she began self-medicating with antiparasitic drugs purchased online without medical supervision, taking these medications for six months. Approximately three months before her psychiatric admission, she was evaluated in an internal medicine department for gastrointestinal and cardiac complaints and also underwent a dermatological assessment. She was diagnosed with Dermographism urticaria and prescribed Levocetirizine 5 mg/day for three months.

The patient had a history of a moderate depressive episode accompanied by anxiety, for which she sought psychiatric care seven years ago in the psychiatric outpatient clinic. She was prescribed Sertraline (50 mg/day) and Alprazolam (0.5 mg twice/day) but did not adhere to the recommended treatment.

Upon admission to the psychiatric unit, a comprehensive evaluation was performed. Physical examination revealed superficial skin lesions secondary to scratching. Neurological examination showed no signs of tremor, cogwheel rigidity, bradykinesia, or other parkinsonian symptoms. Laboratory tests, including a complete blood count and basic metabolic panel, revealed no significant abnormalities. A cranial CT scan performed a year earlier had shown a calcified meningioma in the right cerebellar hemicortex measuring 1.3 x 1 cm, moderately widened cortical sulci and Sylvian fissures, and age-related cerebral atrophy as the general conclusion. The patient’s medical history and paraclinical investigations are presented in Table 1 and Table 2.

Psychological evaluation revealed a Mini Mental Status Examination (MMSE) score of 22/30, with difficulties in calculation and memory tasks, though the patient’s severe hypoacusis may have contributed to challenges in completing the test. On the MADRS (Montgomery–Åsberg Depression Rating Scale) scale, she scored moderately high for depression (32 points), while the PANSS (Positive and Negative Syndrome Scale) total score was 82. On the Hamilton Anxiety Rating Scale (HAM-A), the patient scored 30 points, indicating a moderate level of anxiety with somatic complaints. According to her delusional ideas regarding infestation, the patient presented gastrointestinal complaints, self-reported autonomic symptoms, and sensory and muscular manifestations. Based on her clinical presentation, a diagnosis of Ekbom Syndrome was made, excluding schizophrenia and drug-induced psychotic disorder, as the criteria for these diagnoses were not met.

The patient was started on Risperidone 2 mg every evening, Alprazolam 0.5 mg twice daily, and Zolpidem 10 mg each evening. She was discharged after 15 days, but her delusions and hallucinations persisted, showing no significant improvement.

**Figure 1:**
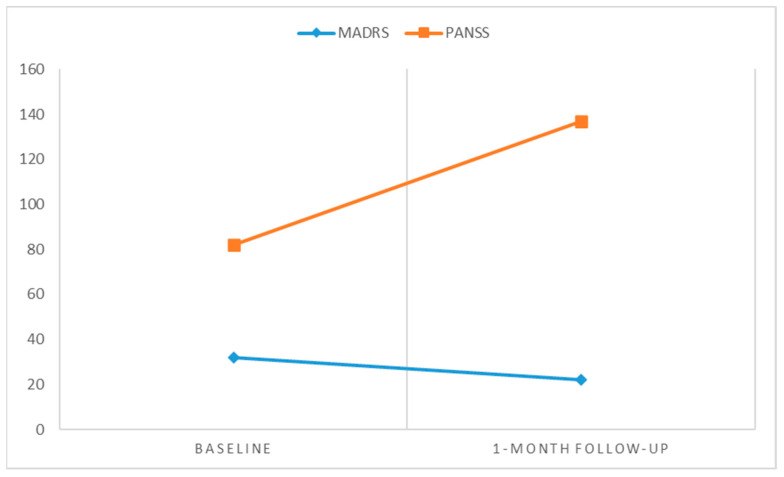
This graphical representation illustrates the evolution of PANSS and MADRS scores in the patient at two time points: admission and one-month follow-up. The PANSS score (orange line) increased from 82 to 137, indicating a worsening of psychotic symptoms under 2 mg of Risperidone (this might suggest poor treatment response or nonadherence to medication). The MADRS Score (blue line) decreased from 32 to 22, reflecting partial improvement in depressive symptoms. However, persistent delusions likely maintained psychological distress.

**Figure 2:**
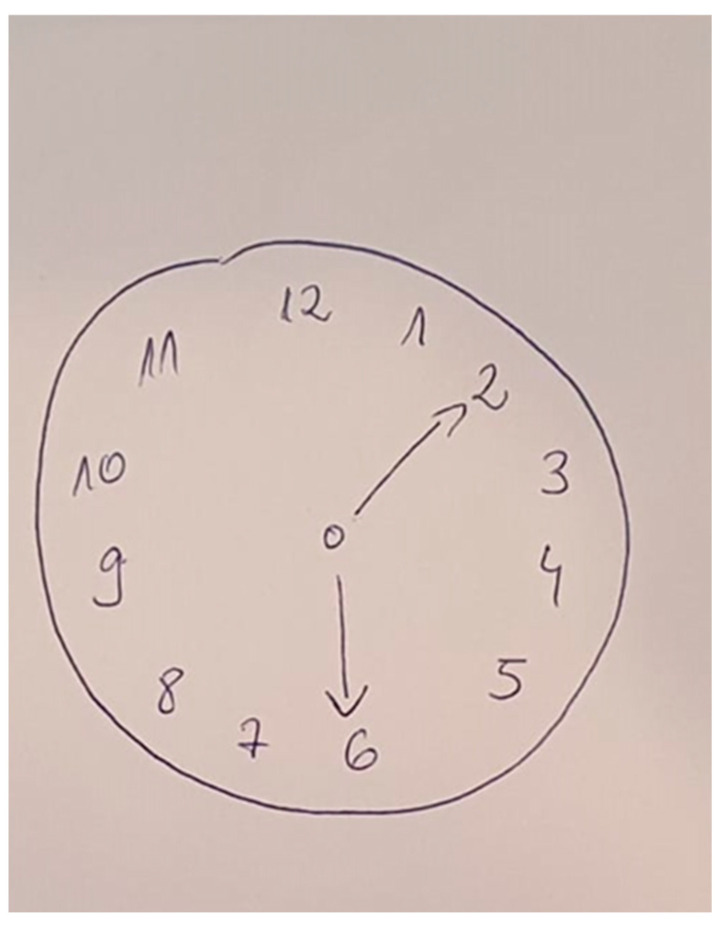
The Clock Drawing Test of the patient. The scoring system awards one point for drawing a closed circle, one point for placing numbers in the correct position, one point for including all 12 correct numbers, and one point for placing hands in the correct positions [8].

She returned for a follow-up visit one month later. During this time, her symptoms remained unchanged in severity compared to her initial hospital admission. A new psychological assessment was performed with the following conclusions: a score of 29/30 on the MMSE, on the State-Trait Anxiety Inventory (State—Y1 form) the patient scored 62 points, indicating a high level of state-level anxiety, on the MADRS scale she had a moderately low (22 points) score for depression, and on the PANSS score she had a total score of 137 (Figure 1). Additionally, the Clock Drawing Test (Sunderland Clock Drawing Test, Figure 2) was conducted to screen for cognitive decline, assess visuo-constructional disorders, and identify any indicators of constructional apraxia. She obtained a perfect score of four out of four, effectively ruling out any of these disorders at the time of evaluation. Risperidone was increased to 4 mg/day; however, after this adjustment, the patient did not return for further evaluation.

## 3. Discussion

Ekbom Syndrome, also known as Delusional Parasitosis, is a rare psychiatric condition with an estimated incidence of 1.9 per 100,000 person-years. Despite its low prevalence, this disorder poses significant challenges in diagnosis and management due to its complex presentation, which often includes persistent somatic delusions and secondary complications such as self-inflicted injuries or poor adherence to medical advice [9,10].

The diagnostic criteria for Ekbom Syndrome are met in this case, as the patient exhibits persistent delusional beliefs of infestation and accompanying visual hallucinations and proprioceptive hallucinations. However, what is particularly noteworthy in this case is the late onset of the disease, as the patient’s symptoms began at an advanced age. If we consider only the American population, a study conducted in central California, funded by the Centers for Disease Control and Prevention, reported a prevalence of 3.7 per 100,000. The study also found that the average age of onset was 52 years, with more than 75% of the cases being female, highlighting the rarity and unusual presentation in our patient [11].

Ekbom Syndrome can manifest in two main forms: primary and secondary. In the primary form, the delusional infestation arises as an isolated mono-delusional disorder and the secondary form occurs as a result of an underlying medical condition. In the present case, the absence of any significant medical or psychiatric comorbidities, as well as the exclusion of conditions such as schizophrenia and drug-induced psychosis, strongly suggests that this is a primary form of Ekbom Syndrome. This distinction is critical for guiding treatment strategies and prognosis [12,13].

The precise mechanism is not fully understood, but it is often associated with dysfunction in the dopaminergic pathways in the brain, particularly hyperactivity of dopamine in the mesolimbic system. This hyperdopaminergic state may lead to the formation of false beliefs and misinterpretations of sensory stimuli. Neurological conditions, such as Parkinson’s disease, traumatic brain injury, or dementia, as well as psychoactive substances, can also contribute to its development. Additionally, Ekbom Syndrome has been linked to decreased serotonergic activity, which can exacerbate the anxiety and obsessive-compulsive traits seen in some patients [5,14].

The primary pharmacological treatment includes antipsychotics, particularly second-generation (atypical) agents such as Risperidone, Olanzapine, and Aripiprazole. These medications are preferred due to their efficacy in managing psychotic symptoms with a lower risk of extrapyramidal side effects compared to first-generation antipsychotics. In cases of severe delusions resistant to treatment, Haloperidol, a first-generation antipsychotic, may be considered. Adjunctive treatments, including selective serotonin reuptake inhibitors (SSRIs), may be used to address comorbid depression or anxiety symptoms when present [15]. In the presented case, antipsychotic treatment was chosen over antidepressants due to the predominance of psychotic symptoms, such as persistent delusions and hallucinations, in the clinical picture. While depressive symptoms were noted, the intensity and impact of the delusions posed a greater risk to the patient’s well-being and functionality. Antipsychotics, particularly those targeting dopamine receptors, have shown efficacy in addressing delusions and hallucinations, which are core features of Ekbom Syndrome. This decision aligns with clinical guidelines emphasizing the need to address the most severe and impairing symptoms first, particularly when psychosis significantly overshadows mood symptoms. Furthermore, second-generation antipsychotics often have mood-stabilizing properties, which may indirectly alleviate depressive symptoms, thus providing a comprehensive approach to treatment [16,17].

A systematic review by McPhie et al. (2022) reported that Risperidone doses between 2–6 mg/day resulted in complete remission in approximately 80% of patients with Ekbom Syndrome. However, a subset of patients required long-term therapy to prevent relapse. [18]. Other reports describe both successes and challenges with Risperidone treatment. In one study, a patient receiving Risperidone (1.5 mg/day) experienced a dramatic improvement within a month but later discontinued medication, leading to symptom recurrence [19]. This pattern of initial improvement followed by relapse upon discontinuation has been observed in multiple cases, suggesting that a minimum treatment duration of three months is necessary to achieve sustained remission. Additionally, it has been noted that adult patients with DP generally require lower doses of antipsychotics than those used in schizophrenia [20].

In other cases, however, Risperidone failed to achieve full remission. One study reported no significant response to Risperidone (2–6 mg/day) or Olanzapine (5 mg/day) over three months, with the patient requiring additional interventions such as hypnotherapy, which also proved ineffective [21]. This highlights the need for individualized treatment strategies, as some patients may require alternative pharmacological approaches.

For this clinical case, treatment was initiated with Risperidone at a dose of 2 mg/day, considering the patient’s advanced age and the presence of multiple comorbidities. The lower initial dose aimed to minimize the risk of side effects, particularly those affecting cardiovascular or metabolic systems, which are common concerns in elderly patients. However, at the one-month follow-up in the outpatient clinic, it became evident that the 2 mg dose was insufficient, as there was no significant improvement in the patient’s symptoms. Based on this evaluation, the dose was titrated to 4 mg/day, adhering to standard protocols for treatment-resistant delusional disorders. Despite this adjustment, a significant challenge in managing this case is the patient’s poor compliance with follow-up visits, a common issue in patients with Delusional Parasitosis. As evidenced by this case, the patient did not return for further evaluation, complicating the monitoring of treatment response and adjustments. This represents a common finding in the literature, as the compliance with treatment is often poor, with follow-up rates averaging 3–5 months before discontinuation [22]. This aligns with our case, where the patient did not return for evaluation after the dose increase. Strategies to improve adherence can include more frequent follow-up appointments, involving caregivers in treatment decisions, home-based monitoring, or community psychiatric services and considering long-acting injectable (LAI) formulations for patients with recurrent nonadherence. The effectiveness of LAI antipsychotics in DP remains underexplored, but their use in schizophrenia suggests potential benefits.

The optimal dose of Risperidone for DP remains undetermined, but reported doses range widely. The present case utilized a dose of 4 mg/day, which falls within the therapeutic range reported in the literature. A comparative analysis of previous cases found that doses from 0.25 mg/day to 5 mg/day have resulted in complete resolution of delusions in some patients, particularly when combined with other agents such as Sertraline or Lithium [23]. According to Freudenmann and Lepping (2008), treatment duration of at least three months is recommended to evaluate the full therapeutic effect of Risperidone in Ekbom Syndrome. In our case, the lack of follow-up after dose escalation prevented long-term assessment of efficacy [20].

Close monitoring is critical, especially in elderly patients, who are at increased risk for side effects such as sedation, metabolic disturbances, or orthostatic hypotension [16]. In this case, the initial dose of 2 mg/day was insufficient to control the symptoms, necessitating an increase in dosage.

## 4. Conclusions

In conclusion, this case report underscores the challenges in treating Ekbom Syndrome, especially in elderly patients with multiple comorbidities and poor compliance. While Risperidone demonstrated its utility as a treatment option, it also revealed limitations at low dosages, as the initial 2 mg/day was insufficient to alleviate the patient’s symptoms, necessitating an increase to 4 mg/day. However, the patient’s lack of adherence to follow-up visits prevented a comprehensive assessment of long-term therapeutic response, a common issue in Delusional Parasitosis management. This underscores the necessity for enhanced monitoring, caregiver involvement, and alternative treatment strategies to improve adherence. Given the heterogeneous response to treatment in Ekbom Syndrome, further research is warranted to establish optimal dosing strategies, the duration of therapy, and adherence-enhancing interventions to ensure sustained clinical improvement in this vulnerable patient population.

## Figures and Tables

**Table 1:** Clinical assessments that summarize the patient’s medical history, including comorbidities and relevant clinical findings.

Clinical Investigations	Clinical Findings	Treatment Recommendations
Dermatological Assessment	Dermographism urticaria	Levocetirizine 5 mg/day for three months
Cardiological Assessment	Hypertension Grade II with High Additional Cardiovascular Risk, Hypertensive and Ischemic Heart Disease, Paroxysmal Atrial Fibrillation Controlled Medically to Sinus Rhythm Angina Pectoris	Amlodipine 5 mg/day Perindopril 5 mg/day Metoprolol 50 mg/day Apixaban 5 mg BID Isosorbide Mononitrate 20 mg BID (if angina symptoms persist) Atorvastatin 40 mg/day
Gastroenterological Assessment	Gastroesophageal Reflux Disease (GERD) Post-Surgical Stomach Chronic Pancreatitis	Omeprazole 40 mg/day or Domperidone 10 mg TID (if needed for motility support)
Pneumological Assessment	Bronchial Asthma	Montelukast 10 mg once daily

**Table 2:** Patient’s paraclinical investigations and relevant laboratory findings.

Paraclinical Investigations	Results
Complete blood count	Eosinophils mildly elevated Slightly low hemoglobin Mildly elevated ESR
Electrolytes	Sodium (Na⁺)—mild fluctuations within normal range Chloride (Cl⁻)—mild fluctuations within normal range
C Reactive Protein	Within normal range
Fasting glucose	Within normal range
Serum creatinine	Within normal range
Liver function	Alanine Aminotransferase slightly elevated Aspartate Aminotransferase slightly elevated Gamma-Glutamyl Transferase (GGT) slightly elevated
Stool specimens	No pathological findings
CT scan	Calcified meningioma in the right cerebellar hemicortex measuring 1.3 × 1 cm, moderately widened cortical sulci and Sylvian fissures and age-related cerebral atrophy

## Data Availability

The data presented in this case report are available on request from the corresponding author due to privacy and ethical restrictions.

## Abbreviations

The following abbreviations are used in this manuscript:

MDPI	Multidisciplinary Digital Publishing Institute
BID	Bis in Die (twice daily)
DP	Delusional Parasitosis
CT	Computed Tomography
ESR	Erythrocyte Sedimentation Rate
IM	Intramuscular
LAI	Long-Acting Injectable
MMSE	Mini Mental State Examination
MADRS	Montgomery–Åsberg Depression Rating Scale
PANSS	Positive and Negative Syndrome Scale
TID	Ter in Die (three times daily)
